# The Burden of Diabetes-Related Chronic Kidney Disease in China From 1990 to 2019

**DOI:** 10.3389/fendo.2022.892860

**Published:** 2022-06-15

**Authors:** Xiaowen Pan, Xiling Lin, Xin Huang, Jingya Xu, Lingxia Ye, Tianyue Zhang, Shaoning Hu, Hongwei Jiang, Yuezhong Ren, Peng-Fei Shan

**Affiliations:** ^1^ Department of Endocrinology and Metabolism, The Second Affiliated Hospital of Zhejiang University School of Medicine, Hangzhou, China; ^2^ Endocrine and Metabolic Disease Center, The First Affiliated Hospital, and College of Clinical Medicine of Henan University of Science and Technology; Medical Key Laboratory of Hereditary Rare Diseases of Henan; Luoyang Sub-Center of National Clinical Research Center for Metabolic Diseases, Luoyang, China; ^3^ Binjiang Institute of Zhejiang University, Hangzhou, China

**Keywords:** chronic kidney disease, diabetes, death, disability-adjusted life years, China

## Abstract

**Objective:**

To analyze the trends in disease burden of diabetes-related chronic kidney disease (CKD) by year, age, gender and types of diabetes in China from 1990 to 2019.

**Methods:**

Data on prevalence, deaths and disability-adjusted life years (DALYs) for diabetes-related CKD were extracted from the Global Burden of Disease (GBD) 2019 study. Analyses were performed by year, age, gender and types of diabetes.

**Results:**

In China, the numbers of deaths and DALYs of diabetes-related CKD continuously increased but the age-standardized rates (per 100,000 population) decreased over 30 years, in which the numbers of deaths and DALYs attributable to type 1 diabetes mellitus (T1DM)-related CKD barely changed and the age-standardized rates decreased over the years; and the number of deaths and DALYs attributable to type 2 diabetes mellitus (T2DM)-related CKD continuously increased, but the age-standardized rates also decreased. In 2019, 76.03 (58.24-95.61) thousand deaths and 2.13 (1.65–2.67) million DALYs were attributable to diabetes-related CKD, of which, T2DM accounted for 83.32% and 77.0% respectively, and T1DM accounted for the rest. Increasing gender disparity was seen, with males being more heavily impacted. The burden of diabetes-related CKD varied among different age groups, with the numbers of deaths and DALYs attributable to T1DM-related CKD peaking between 45 and 54 years of age and T2DM-related CKD peaking between 75 and 79 years of age; and the crude rates of deaths and DALYs attributable to T1DM-related CKD peaking between 70 and 79 years of age and 40 to 54 years of age, respectively, and T2DM-related CKD peaking over 90 years of age. Among neighboring and G20 countries, the burden of diabetes-related CKD in China was relatively controlled reflected by the ranking of adjusted death and DALYs rates.

**Conclusions:**

The burden of diabetes-related CKD in China worsens and shows gender disparities and different age distribution. Greater efforts are needed to improve the health outcomes of these patients, especially among males.

## Introduction

Chronic kidney disease (CKD) is a major cause of mortality and disability and is a leading public health problem worldwide (1, 2). Diabetes mellitus (DM) is considered to have been one of the leading drivers of CKD over the past several decades (1, 3). The International Diabetes Federation (IDF; 10^th^ edition) reported that there were 537 million adults with diabetes in 2021 globally (4). It is estimated that approximately 40% of patients with diabetes will develop diabetic kidney disease (DKD), and approximately 50% of those with DKD may progress to end-stage renal disease (ESRD) during their lifetimes (5, 6). Studies have shown that in the United States of America, DKD is the most common cause of kidney failure, in which 30% of patients with type 1 diabetes mellitus (T1DM) and 40% of patients with type 2 diabetes mellitus (T2DM) develop CKD (7).

Diabetes-related CKD, like CKD due to other causes, is manifested as a low estimated glomerular filtration rate (eGFR) and an elevated urinary albumin excretion rate (AER) and is associated with increased rates of all-cause and cardiovascular disease (CVD)-related mortality (2, 8–10). CKD is a risk multiplier for diabetes and aggravates the burden of diabetes; in addition, both CKD and diabetes are strongly associated with CVD (5, 6). Furthermore, diabetes-related CKD is a principal cause of progression to ESRD, which requires costly renal replacement therapy, including dialysis or renal transplantation (5, 11, 12).

China has a considerable burden of diabetes. 141 million adults were living with diabetes accounting for 25% of all adults living with diabetes worldwide, being at risk of serious complications like CKD (4). Additionally, instead of glomerulonephritis-related CKD, diabetes-related CKD took over the main cause of CKD in China recently (13). The treatment of CKD costs, in which the cost of dialysis reaches 106 billion Renminbi (RMB; about 15.3 billion dollars) every year (14). The long-term impact of diabetes-related CKD on people’s health and the country’s economy should be paid much more attention and taken actions to relieve the disease burden. However, few studies have analyzed the comprehensive burden of diabetes-related CKD in China to our knowledge. The specific distribution of diabetes-related CKD in China and the influence of gender disparity and aging on the disease burden warrant an in-depth investigation that will inform healthcare policies and interventions. Therefore, this study aimed to assess the global trend of diabetes-related CKD in China, by using the latest death and disability-adjusted life years (DALYs) data from the Global Burden of Disease study (GBD) 2019 study.

## Methods

### Data Source

The GBD is unique in its approach to generating estimates including incidence, prevalence, deaths, and DALYs for all regions by using all available data from administrative hospital and medical claims records, cause of death records, the published and unpublished literature (15). The health loss due to CKD has been quantified by deaths and DALYs in this GBD study. DALYs are defined as the sum of years lived with disability and years of life lost owing to premature death, and are used to fully assess the impact of both the fatal and nonfatal outcomes of diseases (16). We quantified the burden of diabetes-related CKD during the period from 1990 to 2019. The following data on diabetes-related CKD were collected from the Global Health Data Exchange: (1) Gender-specific data on prevalence, deaths and DALYs as absolute numbers and age-standardized rates (per 100,000 population), annually from 1990 to 2019 in China; (2) Gender- and age-specific data on deaths and DALYs as absolute numbers and crude (i.e., unadjusted) rates in 2019 in China; (3) National data on deaths and DALYs as age-standardized rates in 2019. Ethics approval and informed consent were not required for this study because of the use of publicly accessible data.

### Statistical Analysis

Data are expressed as absolute values with 95% uncertainty intervals (UI). The rates of deaths and DALYs are expressed as the number per 100,000 population. All statistical analyses were conducted using Prism software (*version* 8; GraphPad), except where otherwise specified.

## Results

### Trends and Gender Disparity of Diabetes-Related CKD in China

In China, the numbers of people living with diabetes continuously increased from 35.49 (31.95-39.41) million in 1990 to 91.98 (84.23-100.52) million in 2019 by 159.17%. The age-standardized rates per 100,000 population of prevalence due to diabetes increased from 3740.02 (3381.06-4128.84) in 1990 to 4613.09 (4215.52-5047.67) in 2019 (**Figures S1A, B** **)**. The numbers of people with diabetes-related CKD also increased over 30 years, from 17.34 (15.54-19.42) million (accounting for 48.86% of total population with diabetes) in 1990 to 31.65 (28.65-34.57) million (accounting for 34.41% of total population with diabetes) in 2019. The age-standardized rates of prevalence increased from 29.79 (25.59-34.58) in 1990 to 39.07 (33.62-45.50) in 2019 in T1DM-related CKD and decreased from 1678.25 (1523.94-1851.42) in 1990 to 1606.88 (1457.70-1760.75) in 2019 in T2DM-related CKD (**Figures S1C, D** **)**.

In the diabetes-related CKD, the absolute numbers of deaths and DALYs have both shown increasing trends over the past 30 years. Death numbers were 38.60 (29.74-48.21) thousand in 1990 and 76.03 (58.24-95.61) thousand in 2019 increasing by 96.97%, and DALYs number were 1.36 (1.03-1.71) million in 1990 and 2.13 (1.65–2.67) million in 2019 increasing by 56.62%, respectively (**Figures S1E, F** **)**.

In 2019, death numbers of T1DM-related CKD were 12.68 (8.46-18.33) thousand and T2DM-related CKD were 63.35 (49.79-77.28) thousand, which accounted for 16.68% and 83.32%, respectively. DALYs numbers of T1DM-related CKD were 0.49 (0.33-0.69) million and T2DM-related CKD were 1.64 (1.31-1.98) million, which accounted for 23.0% and 77.0%, respectively.

**Figure 1:**
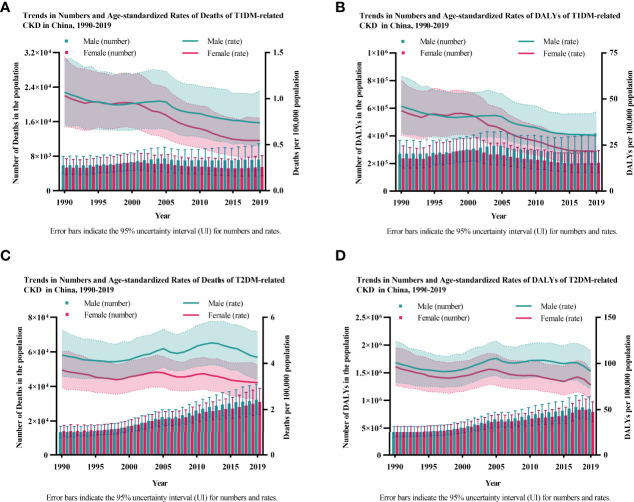
The burden of diabetes-related CKD by year from 1990 to 2019. **(A, B)** show the numbers and age-standardized rates of deaths and DALYs of T1DM-related CKD. **(A)** Deaths; **(B)** DALYs; **(C, D)** show the numbers and age-standardized rates of deaths and DALYs of T2DM-related CKD. **(C)** Deaths; **(D)** DALYs; CKD, chronic kidney disease; T1DM, type 1 diabetes mellitus; T2DM, type 2 diabetes mellitus; DALYs, disability-adjusted life-years.

When described individually, the numbers of deaths and DALYs attributable to T1DM-related CKD barely changed or increased slightly (Death 1990: female 5.33 (3.69-7.41) thousand, male 5.86 (3.94-7.94) thousand; Death 2019: female 5.47 (3.50-8.17) thousand, male 7.21 (4.67-10.85) thousand; DALYs 1990: female 237.51 (166.98-327.86) thousand, male 270.79 (183.25-367.47) thousand; DALYs 2019: female 205.56 (136.50-293.06) thousand, male 288.12 (192.46-415.10) thousand); and the age-standardized rates of deaths and DALYs decreased over the years in both females and males [Death 1990: female 1.03 (0.70-1.46), male 1.07 (0.72-1.43); Death 2019: female 0.54 (0.36-0.79), male 0.74 (0.49-1.09); DALYs 1990: female 43.68 (30.24-60.42), male 46.06 (31.51-62.41); DALYs 2019: female 21.59 (14.73-29.81), male 30.25 (20.71-43.12)] (**Figures 1A, B**). However, in T2DM-related CKD, the number of deaths and DALYs continuously increased (Death 1990: female 14.14 (10.91-17.47) thousand, male 13.27 (10.00-16.93) thousand; Death 2019: female 31.06 (23.72-38.91) thousand, male 32.30 (24.27-41.39) thousand; DALYs 1990: female 429.00 (335.32-530.61) thousand, male 420.05 (317.21-528.13) thousand; DALYs 2019: female 792.93 (617.20-972.75) thousand, male 847.48 (655.07-1064.27) thousand), and the age-standardized rates of deaths and DALYs decreased but there were fluctuations in both two genders [Death 1990: female 3.71 (2.94-4.53), male 4.37 (3.44-5.45); Death 2019: female 3.17 (2.43-3.95), male 4.27 (3.28-5.40); DALYs 1990: female 96.43 (76.25-117.21), male 100.95 (79.20-124.17); DALYs 2019: female 76.62 (60.34-93.78), male 91.64 (71.98-113.97)] (**Figures 1C, D** **)**.

We also noticed gender disparity in both T1DM- and T2DM-related CKD. Although there were a few years when the female had higher age-standardized deaths and DALYs rates than males in T1DM-related CKD, males bore a severer disease burden than females both in numbers and rates of deaths and DALYs overall. And this disparity was enlarged in the recent 15 years (**Figures 1A–D**).

### Trends of Diabetes-Related CKD by Age in China

Considering the wide difference in the burden of T1DM-related and T2DM-related CKD in China, we investigated the age-specific burden separately.

**Figure 2:**
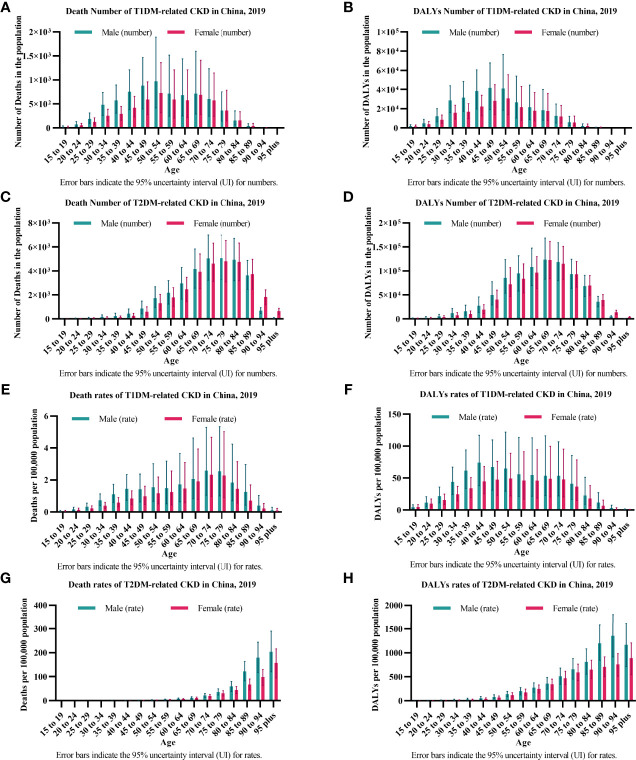
The burden of diabetes-related CKD by age in 2019. **(A, B)** show the numbers of deaths and DALYs of T1DM-related CKD. **(A)** Deaths; **(B)** DALYs; **(C, D)** show the numbers of deaths and DALYs of T2DM-related CKD. **(C)** Deaths; **(D)** DALYs; **(E, F)** show the crude rates of deaths and DALYs of T1DM-related CKD. **(E)** Deaths; **(F)** DALYs; **(G, H)** shows the crude rates of deaths and DALYs of T2DM-related CKD. **(G)** Deaths; **(H)** DALYs; CKD, chronic kidney disease; T1DM, type 1 diabetes mellitus; T2DM, type 2 diabetes mellitus; DALYs, disability-adjusted life-years.

In T1DM-related CKD, the numbers of deaths and DALYs peaked between 45 and 54 years of age [Death number: female 0.73 (0.32-1.36) thousand, male 0.97 (0.42-1.89) thousand; DALYs number: female 30.67 (13.79-55.38) thousand, male 41.53 (19.19-67.75) thousand] (**Figures 2A, B** **)**. The crude rates of deaths peaked between 70 and 79 years of age [Death rate: female 2.33 (0.97-4.66), male 2.58 (1.04-5.28)] and DALYs peaked between 40 and 54 years of age [DALYs rate: female 49.26 (22.15-88.94), male 73.85 (37.96-116.77)] (**Figures 2E, F** **)**. The gender disparity was severe between 25 and 54 years of age with males bearing more disease burden both in deaths and DALYs and gradually narrowing over 54 years of age (**Figures 2A, B, E, F**).

In T2DM-related CKD, the numbers of deaths and DALYs peaked between 75 and 79 years of age (Death number: female 4.82 (3.09-6.53) thousand, male 5.07 (3.28-6.99) thousand) and 65-69 years of age (DALYs number: female 122.68 (83.91-161.64) thousand, male 123.94 (82.98-168.45) thousand), respectively (**Figures 2C, D** **)**. The crude rates of deaths and DALYs peaked over 90 years of age [Death rate: female 157.71 (94.48-216.02), male 204.52 (120.76-290.08); DALYs rate: female 891.33 (544.59-1206.58), male 1353.33 (914.46-1807.69)] (**Figures 2G, H** **)**. The gender disparity was distinct between 30 and 64 years of age with males bearing more disease burden both in the numbers of deaths and DALYs and gradually narrowing over 64 years of age and was distinct over 85 years of age in the crude rates of deaths and DALYs (**Figures 2C, D, G, H**).

### Burden of Diabetes-Related CKD in China and Neighboring Countries and G20 Countries

To better evaluate the disease development in China, we compared the disease burden in China and neighboring countries, and G20 countries.

**Figure 3:**
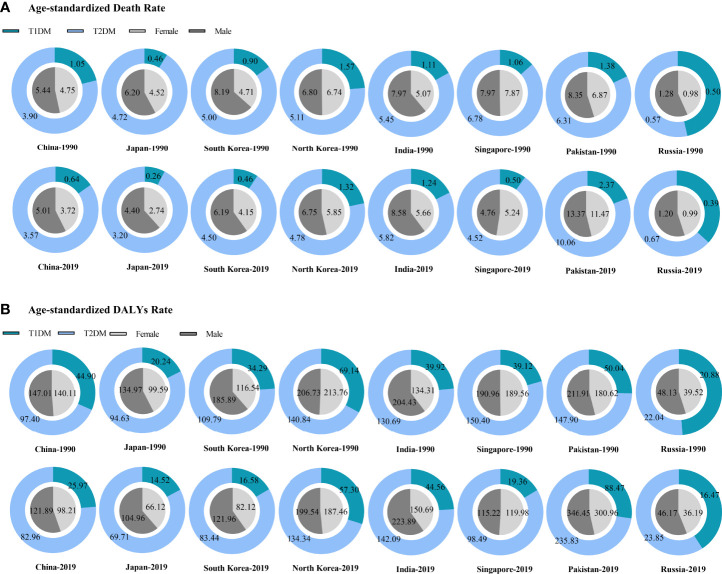
The burden of diabetes-related CKD in China and neighboring countries. **(A)** Age-standardized death rate; **(B)** Age-standardized DALYs rate. CKD, chronic kidney disease; T1DM, type 1 diabetes mellitus; T2DM, type 2 diabetes mellitus; DALYs, disability-adjusted life-years.

When compared to neighboring countries, especially paying attention to different types of diabetes and genders, we found most of them including China, which tend to be that T2DM-related CKD had higher age-standardized death and DALYs rates, and males bore the severer burden (except for North Korea and Singapore where gender disparity was not obvious) in both 1990 and 2019. Over 30 years, the age-standardized rates of deaths and DALYs decreased in China and in most of the neighboring countries except for India and Pakistan which still had a considerable increase (**Figure 3**).

When compared to G20 countries, a much wider range in the world, the specific numbers and rates of DALYs were listed and ranked, to better see the status of China in the burden of diabetes-related CKD (**Tables 1**, **2**).

**Table 1:** All-age DALYs and age-standardized DALYs rate (per 100,000 population) for T1DM-related CKD in 1990 and 2019 among the G20 countries (the 20th member is the European Union).

Country	All-age DALYs total (thousand) (95%UI)							Age-standardized DALYs rate per 100K population (95%UI)		
	1990	Rank	2019	Rank	Change (%)	1990	rank	2019	Rank	Change (%)
America	16.99 (12.18-22.68)	9	48.18 (32.80-66.92)	6	183.58	26.14 (18.46-35.16)	11	41.39 (28.42-56.86)	6	58.34
Argentina	11.71 (7.63-16.57)	12	17.77 (11.50-25.19)	12	51.78	36.38 (23.70-51.44)	9	35.38 (22.99-50.31)	7	-2.76
Australia	0.81 (0.64-1.06)	19	1.79 (1.20-2.58)	19	120.88	4.34 (3.38-5.73)	19	5.64 (3.79-8.11)	18	29.82
Brazil	44.95 (31.84-59.74)	4	72.08 (49.48-99.56)	5	60.35	37.80 (26.40-50.86)	8	29.19 (20.26-40.00)	8	-22.78
Canada	2.48 (1.68-3.46)	18	2.78 (2.58-5.25)	18	52.02	7.94 (5.38-11.05)	15	7.40 (4.93-10.27)	14	-6.82
China	508.30 (362.79-673.71)	1	493.68 (334.38-689.36)	2	-2.88	44.90 (31.77-59.83)	4	25.97 (18.30-35.12)	9	-42.15
England	3.58 (2.41-5.05)	17	3.85 (2.60-5.41)	17	7.54	5.78 (3.98-7.95)	18	4.84 (3.32-6.52)	19	-16.31
France	4.26 (2.91-6.19)	16	6.09 (4.20-8.53)	16	43.02	5.95 (4.11-8.52)	17	6.30 (4.25-8.88)	16	5.89
Germany	7.86 (5.04-11.62)	13	10.98 (7.12-16.80)	14	39.65	7.24 (4.80-10.47)	16	7.29 (4.91-10.32)	15	0.57
India	250.67 (160.05-374.38)	2	588.07 (364.10-888.58)	1	134.60	39.92 (25.04-59.91)	7	44.56 (27.47-67.69)	5	11.61
Indonesia	168.81 (125.81-217.18)	3	253.44 (182.58-339.67)	3	50.13	105.63 (77.40-138.28)	1	89.51 (64.95-119.38)	2	-15.3
Italy	6.48 (4.31-9.36)	14	6.12 (4.12-8.55)	15	-5.6	8.49 (5.77-11.99)	14	6.06 (4.16-8.29)	17	-25.6
Japan	33.04 (21.50-46.88)	6	29.78 (19.03-42.95)	8	-9.88	20.24 (13.47-28.43)	13	14.52 (9.44-20.69)	13	-28.26
Mexico	31.39 (22.76-41.27)	7	158.54 (101.65-226.77)	4	405.05	52.29 (36.55-70.63)	3	122.87 (79.15-175.55)	1	135.00
Russia	36.03 (26.18-49.49)	5	33.06 (23.38-45.94)	7	-8.25	20.88 (15.15-28.50)	12	16.47 (11.89-22.44)	12	-21.11
Saudi Arabia	5.35 (3.20-8.32)	15	21.55 (13.29-33.77)	11	302.58	55.26 (32.39-87.84)	2	57.29 (35.54-89.51)	3	3.67
South Africa	12.44 (8.13-18.05)	11	25.66 (16.89-37.17)	9	106.35	43.47 (28.75-63.73)	5	46.64 (30.56-67.69)	4	7.28
South Korea	14.21 (10.24-18.62)	10	13.55 (8.91-18.65)	13	-4.64	34.29 (24.06-45.40)	10	16.58 (11.51-22.30)	11	-51.65
Turkey	19.29 (12.61-27.90)	8	22.02 (14.50-31.57)	10	14.14	42.24 (26.84-61.95)	6	23.41 (15.46-33.42)	10	-44.59

CKD, chronic kidney disease; T1DM, type 1 diabetes; DALYs, disability-adjusted life years; 95% UI, 95% uncertainty interval.

**Table 2:** All-age DALYs and age-standardized DALYs rate (per 100,000 population) for T2DM-related CKD in 1990 and 2019 among the G20 countries (the 20th member is the European Union).

Country	All-age DALYs total (thousand) (95%UI)							Age-standardized DALYs rate per 100K population (95%UI)		
	1990	Rank	2019	Rank	Change (%)	1990	rank	2019	Rank	Change (%)
America	533.44 (430.32-631.65)	3	2026.44 (1647.08-2412.62)	1	279.88	87.55 (70.61-103.98)	12	159.54 (130.30-189.70)	5	82.24
Argentina	45.88 (35.90-55.32)	10	87.63 (68.84-106.33)	10	91.01	142.57 (112.64-171.54)	5	161.92 (126.91-196.85)	4	13.58
Australia	2.99 (2.34-3.82)	19	10.07 (7.53-13.08)	19	236.72	15.55 (12.29-19.62)	19	23.43 (17.60-30.15)	18	50.72
Brazil	96.30 (77.50-114.87)	6	245.48 (199.78-291.25)	7	154.92	106.69 (86.65-126.43)	8	103.75 (84.78-122.40)	9	-2.75
Canada	13.28 (10.18-16.56)	18	28.43 (21.82-36.01)	17	114.06	40.73 (31.44-50.58)	14	40.65 (31.08-51.40)	14	-0.19
China	849.04 (663.74-1032.54)	1	1640.41 (1312.35-1979.87)	3	93.21	97.40 (78.35-116.36)	10	82.96 (66.87-99.00)	11	-14.83
England	18.02 (13.92-22.96)	17	23.96 (18.59-30.34)	18	32.93	23.53 (18.20-29.63)	17	22.34 (17.26-28.36)	19	-5.03
France	24.09 (18.36-30.86)	14	37.49 (28.34-48.69)	16	55.62	28.02 (21.46-35.40)	16	25.05 (19.20-32.29)	16	-10.61
Germany	58.38 (45.60-73.23)	9	131.19 (100.60-171.01)	9	124.69	45.22 (35.14-56.58)	13	61.25 (47.37-78.60)	13	35.46
India	601.24 (446.10-773.11)	2	1648.77 (1227.57-2117.24)	2	174.23	130.69 (99.62-163.89)	6	142.09 (107.11-180.96)	8	8.72
Indonesia	203.04 (157.20-249.58)	4	428.02 (329.03-539.66)	5	110.81	178.25 (141.50-214.97)	4	183.20 (144.46-225.82)	3	2.78
Italy	34.68 (26.80-43.78)	12	47.25 (36.37-60.63)	15	36.22	38.52 (29.78-47.92)	15	29.70 (23.05-37.50)	15	-22.89
Japan	157.74 (131.52-182.38)	5	257.02 (208.19-304.58)	6	62.94	94.63 (79.33-109.41)	11	69.71 (56.93-83.23)	12	-26.34
Mexico	80.04 (65.58-94.20)	8	487.38 (383.52-604.96)	4	508.96	186.60 (155.67-217.85)	3	410.66 (324.66-508.17)	1	120.08
Russia	39.64 (29.94-51.49)	11	55.78 (42.33-72.03)	14	40.72	22.04 (16.76-28.21)	18	23.85 (18.18-30.47)	17	8.21
Saudi Arabia	18.63 (13.10-25.20)	16	60.77 (44.19-81.14)	13	226.24	324.27 (236.09-439.53)	1	349.64 (261.69-456.77)	2	7.82
South Africa	21.77 (16.45-27.70)	15	67.45 (51.75-83.85)	12	209.80	101.51 (77.65-127.18)	9	154.13 (119.37-188.18)	7	51.83
South Korea	32.85 (27.69-37.32)	13	74.26 (64.33-84.42)	11	126.06	109.79 (94.08-123.80)	7	83.44 (72.36-94.89)	10	-33.81
Turkey	82.14 (62.03-112.71)	7	138.08 (107.16-174.81)	8	68.11	234.68 (176.43-324.60)	2	159.32 (124.36-201.06)	6	-32.11

CKD, chronic kidney disease; T2DM, type 2 diabetes; DALYs, disability-adjusted life years; 95% UI, 95% uncertainty interval.

In T1DM-related CKD, China was once ranked 1^st^ in 19 countries in the DALYs number in 1990 and then declined to 2^nd^ in 2019 replaced by India. And the age-standardized rates of DALYs were also declined to 9^th^ in 2019 from 4^th^ in 1990. What caught our eyes was the tremendous increase in DALYs in America and Mexico in 2019 compared to 1990 (**Table 1** **)**.

In T2DM-related CKD, China was also ranked 1^st^ in the DALYs number in 1990 and then declined to 3^rd^ in 2019 replaced by America. And the age-standardized rates of DALYs were also declined to 11^th^ in 2019 from 10^th^ in 1990. Consistent with the changes in T1DM-related CKD, America, and Mexico bore the seriously increased burden in both numbers and age-standardized rates of DALYs as well (**Table 2**).

### Trends in Leading Causes of CKD in China

**Figure 4:**
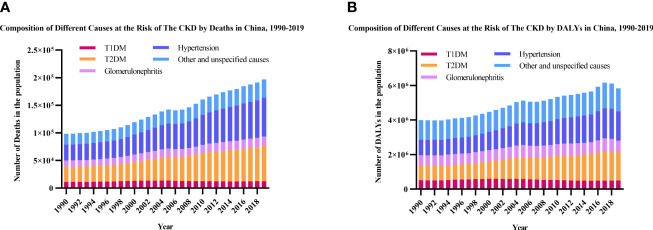
Leading causes of CKD by year. **(A)** Number of deaths; **(B)** Number of DALYs. CKD, chronic kidney disease; T1DM, type 1 diabetes mellitus; T2DM, type 2 diabetes mellitus; DALYs, disability-adjusted life-years.

During the past three decades, hypertension, T2DM, and “other and unspecified causes” were the three leading causes of deaths and DALYs among people with CKD (**Figure 4**). The death number of T1DM-related CKD accounted for 11.34% in total CKD in 1990 and decreased to 6.44% in 2019, while T2DM-related CKD accounted for 27.80% in total CKD in 1990 and increased to 32.20% in 2019. The DALYs number of T1DM-related CKD accounted for 12.72% in total CKD in 1990 and decreased to 8.46% in 2019, while T2DM-related CKD accounted for 21.24% in total CKD in 1990 and increased to 28.13% in 2019. The major cause of CKD remained hypertension and T2DM over 30 years, and the proportion of glomerulonephritis, other and unspecified causes were decreasing.

## Discussion

This study comprehensively demonstrated the trends in the deaths and DALYs of diabetes-induced CKD by age, gender and types of diabetes in China from 1990 to 2019. The numbers of deaths and DALYs both showed increasing trends in total diabetes-related CKD where T1DM-related CKD changed a little and T2DM-related CKD increased obviously. The age-standardized rates decreased in both T1DM- and T2DM-related CKD in 2019 as compared to 1990. Gender disparity in the burden of diabetes-related CKD was widened over the past three decades, with males bearing a greater burden in terms of both deaths and DALYs since 1990. In the aging process, T1DM- and T2DM-related CKD displayed different distribution in both deaths and DALYs. When putting China in perspective more international, evaluated by age-standardized death and DALYs rates, China had some progress but still bore a serious burden. Over 30 years, hypertension and T2DM still were leading causes of CKD in China.

What is worth to be paid attention to is that T1DM-related CKD accounted for 1.8% of the prevalence number of diabetes-related CKD, however, it accounted for 16.7% and 23.0% of all deaths and DALYs due to diabetes-related CKD in 2019. The lower prevalence of T1DM-related CKD along with the disproportionately high death and DALYs rates may be partly explained by the higher proportion of deaths and DALYs below the age of 50 in T1DM patients than in T2DM patients. In addition, glycemic control is tightly associated with the development and progression of diabetes-related CKD (17–21). People with T1DM need daily injections of insulin to control their blood glucose levels. Although in recent years, China has gradually shifted from traditional mode to intensive mode which adapted the insulin analogs and the insulin pens and pumps to treat T1DM, the proportions of intensive treatment are still lower in China than in other countries (22); besides, T1DM frequently occurs in childhood (4), it’s relatively difficult for children to have self-treatment and dietary adherence. The duration of pubertal diabetes may play a role in accelerating renal damage (23). The burden of T1DM-related CKD in China was still severe but the numbers of deaths and DALYs remained relatively stable over 30 years. One of the reasons should be effective pediatric diabetes education with the support of the project of Standardized Management of Children with Diabetes and the Chinese Children’s Diabetes Collaborative Group (22). The growing burden of T2DM-related CKD may be due to the increasing burden of T2DM in China, which accounts for 90% of all diabetes and has an increasing trend mainly due to dietary changes and physical inactivity brought by urbanization (24); moreover, CKD is more prevalent in T2DM patients due to their prolonged life expectancy (4, 25–27). The age-standardized rates of both T1DM- and T2DM-related CKD were declined over decades, contrary to the rates globally which increased in 2019 compared to 1990 (3), indicating that the overall increase in the numbers in China might have been partly driven largely by population growth.

The results of the analysis by age revealed a single-peak distribution of the number and crude rates of deaths and DALYs in both types of diabetes. The greatest numbers and crude rates of both measurements were found happening much earlier in T1DM-related CKD than in T2DM-related CKD. This may be due to that T1DM is more common in younger people, and in contrast, T2DM is more common among adults. The earlier peak of deaths and DALYs in T1DM-related CKD may be a result of the earlier and longer course of the disease.

Based on the latest data from the GBD 2019 study, we also revealed a gender-specific pattern: overall, males suffered more than females, as measured by the numbers and rates of deaths and DALYs. Physical, biological, and behavioral factors, socioeconomic status, educational status, and social disparities contribute to the differences in the deaths and DALYs due to diabetes-related CKD between two genders (28, 29). The higher prevalence of CKD stages 1–3 in females and higher mortality in males suggest that women had a lower risk of CKD progression and deaths as compared with men (2). This may be due to gender differences in health behaviors such as smoking and alcohol use, and the effect of sex hormones, where estrogens may have a protective effect on the vasculature, while testosterone has a deleterious effect (30, 31). Furthermore, women had increased NO availability and reduced renal oxidative stress when compared to men, which might have protective effects against the progression of diabetes-related CKD (32).

When compared to neighboring countries and G20 countries, China made some progress rather than continues to deteriorate reflected in rates of deaths and DALYs. India and Pakistan stood severer burden in 2019 than in 1990. As mentioned above, diabetes-related CKD is the most common cause of ESRD, which is a lethal threat in many parts of the world where dialysis is not available or affordable (6). However, kidney-care services like dialysis, renal transplantation, and other kidney medications were hard to have accessibility and affordability for many families. Fortunately, the Chinese government has initiated universal health insurance coverage to solve this problem since 2003 (33) and set up more than 150 independent hemodialysis centers since 2016 (14). It brings great hope for thousands of families with diabetes-related CKD.

In conclusion, this study revealed the burden of diabetes-related CKD in China, both in terms of deaths and DALYs. Gender disparity existed in the burden of diabetes-related CKD, with males being more heavily affected than females. Preventive efforts should be targeted on different genders. The specific characteristics of T1DM and T2DM bring different features of related CKD like age distribution, and attention should be paid to different age groups, respectively. The prevention and treatment of diabetes are also very important. Several limitations of this study should be mentioned. First, In the GBD study, the causes of CKD included T1DM, T2DM, hypertension, glomerulonephritis and “other and unspecified causes”. The burden of hypertension-related CKD was also serious in China. We mainly focused on the diabetes-related CKD, so the burden of hypertension-related CKD was not analyzed in this study. When distinguishing the main cause of CKD clinically, the disease history, course and complications will help define the exactly cause. In the GBD, the causes of CKD that T1DM and T2DM were determined on the basis of ICD10 (E10.2-E10.29) and (E11.2-E11.29), respectively. However, CKD was often caused by comorbid conditions, such as diabetes and hypertension which are both closely linked to CKD progression, which may lead to uncertainty about the definite underlying cause of CKD (2). Secondly, there was no more detailed data from provinces of China, only the data of total burden of diabetes-related CKD in the Chinese Mainland (not including Taiwan) were analyzed. Thirdly, as the GBD study updates annually, further exploration and longer-term surveillance of diabetes-related CKD is required.

## Data Availability Statement

The original contributions presented in the study are included in the article/**Supplementary Material**. Further inquiries can be directed to the corresponding authors.

## Author Contributions

XP, XL and XH, and conceived the study, collected data, performed the statistical analysis and participated in writing and preparation of the report. JX, SH, LY, TZ were involved in the data collection, interpretation of the data and preparation of the report. P-FS, and YR, and HJ designed and coordinated the study, acquired funding, performed the statistical analysis and participated in writing and editing the final report. P-FS assumes full responsibility for the overall content of this report. All authors contributed to the article and approved the submitted version.

## Funding

This work was supported by grants from the National Natural Science Foundation of China (grant numbers 81870564 to P-FS).

## Conflict of Interest

The authors declare that the research was conducted in the absence of any commercial or financial relationships that could be construed as a potential conflict of interest.

## Publisher’s Note

All claims expressed in this article are solely those of the authors and do not necessarily represent those of their affiliated organizations, or those of the publisher, the editors and the reviewers. Any product that may be evaluated in this article, or claim that may be made by its manufacturer, is not guaranteed or endorsed by the publisher.

## Supplementary Material

Supplementary Figure 1 The burden of diabetes and diabetes-related CKD in China by year from 1990 to 2019. **(A, B)** show the prevalence of diabetes in China. **(A)** The numbers of prevalence; **(B)** The age-standardized rates of prevalence; **(C, D)** show the prevalence of diabetes-related CKD in China. **(C)** The numbers of prevalence; **(D)** The age-standardized rates of prevalence; **(E, F)** show the numbers of deaths and DALYs due to diabetes-related CKD in China. **(E)** Deaths; **(F)** DALYs. CKD, chronic kidney disease; T1DM, type 1 diabetes mellitus; T2DM, type 2 diabetes mellitus; DALYs, disability-adjusted life-years.
