# Supplementary figures and images for: The Burden of Diabetes-Related Chronic Kidney Disease in China From 1990 to 2019
Source: Front Endocrinol (Lausanne). 2022 Jun 15;13:892860. doi: 10.3389/fendo.2022.892860 (PMC9240757; doi:10.3389/fendo.2022.892860)

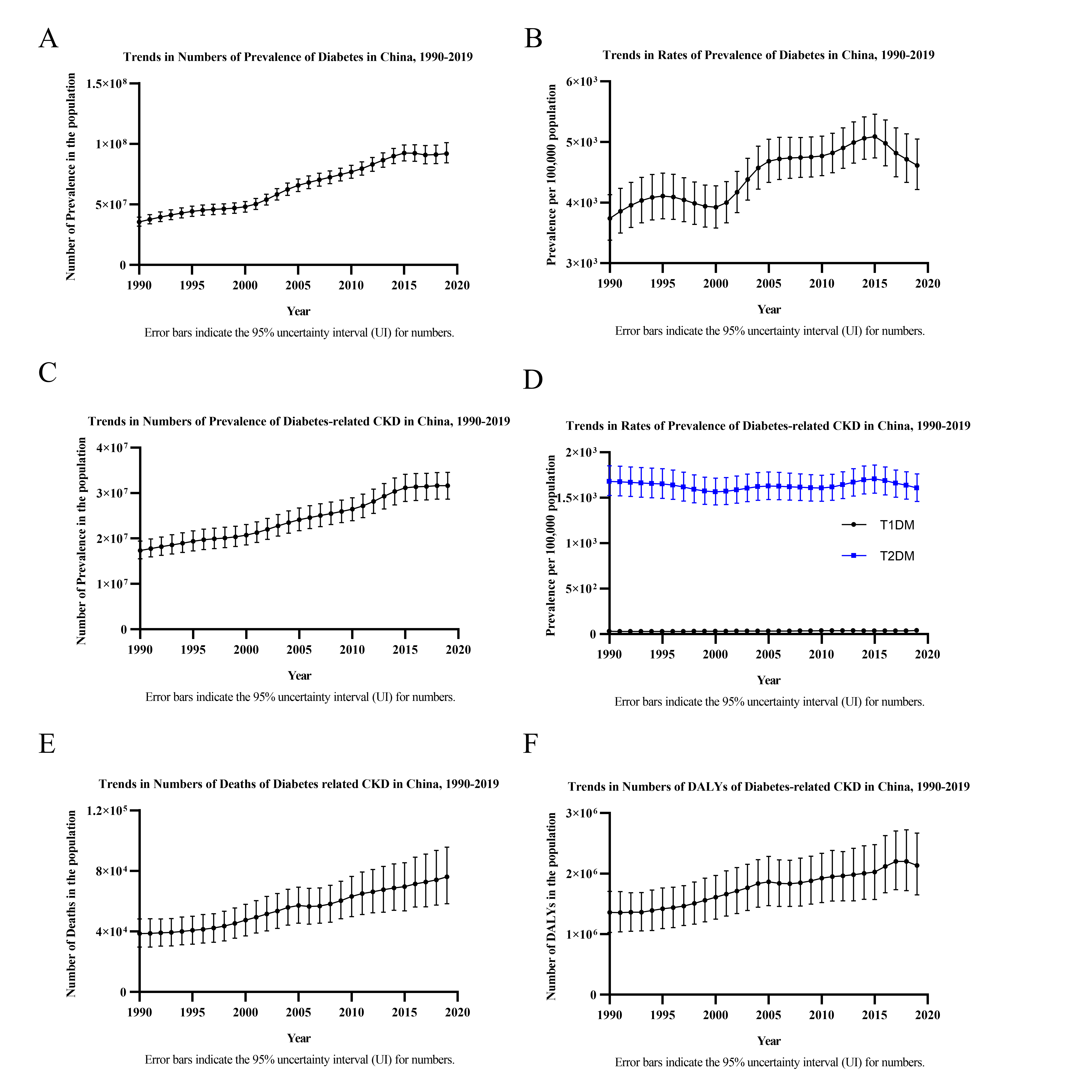

Supplement: Supplementary Figure 1 — The burden of diabetes and diabetes-related CKD in China by year from 1990 to 2019. (A, B) show the prevalence of diabetes in China. (A) The numbers of prevalence; (B) The age-standardized rates of prevalence; (C, D) show the prevalence of diabetes-related CKD in China. (C) The numbers of prevalence; (D) The age-standardized rates of prevalence; (E, F) show the numbers of deaths and DALYs due to diabetes-related CKD in China. (E) Deaths; (F) DALYs. CKD, chronic kidney disease; T1DM, type 1 diabetes mellitus; T2DM, type 2 diabetes mellitus; DALYs, disability-adjusted life-years. [file Image_1.tif]
